# A Species-Independent Lateral Flow Test to Detect Rift Valley Fever Virus Antibodies Using a Double Antigen Approach

**DOI:** 10.3390/v18030316

**Published:** 2026-03-03

**Authors:** Paul J. Wichgers Schreur, Heleen de Vogel-van den Bosch, Ruben Massop, José Harders-Westerveen, Sandra van de Water, Barry Rockx, Aart van Amerongen

**Affiliations:** 1 Department of One Health Virology, Wageningen Bioveterinary Research, Wageningen University & Research, Houtribweg 39, 8221 RA Lelystad, The Netherlandsbarry.rockx@wur.nl (B.R.); 2 BioSensing & Diagnostics, Wageningen Food & Biobased Research, Wageningen University & Research, Bornse Weilanden 9, 6708 WG Wageningen, The Netherlands; heleen.vandenbosch@wur.nl (H.d.V.-v.d.B.); aart.vanamerongen@wur.nl (A.v.A.)

**Keywords:** rift valley fever virus, lateral flow test, double-antigen, ELISA, bunyavirus, pen-side test

## Abstract

Rift Valley fever virus (RVFV) is a re-emerging, vector-borne pathogen endemic to Africa and the Arabian Peninsula, posing an increasing threat to human and animal health. Outbreaks have severe economic and social impacts on farmers, communities, and governments. Current diagnostic methods rely on PCR and ELISA; however, rapid pen-side tests would enable faster, cost-effective monitoring and outbreak control. Here, a species- and immunoglobulin class-independent capillary flow immunodiagnostic assay (lateral flow test; LFT) for detecting RVFV-specific antibodies is described. The assay uses a double-antigen approach, coupling the RVFV nucleocapsid protein, a major viral antigen, both to carbon nanoparticles and to a nitrocellulose membrane. The method was qualified with immune sera from sheep, calves, goats, and humans and benchmarked against a newly developed double-antigen ELISA and a commercial competition ELISA. Both the LFT and double-antigen ELISA demonstrated high specificity and sensitivity. This advancement brings RVFV-specific pen-side testing significantly closer to practical implementation.

## 1. Introduction

Rift Valley fever virus (RVFV) is a zoonotic, mosquito-borne bunyavirus belonging to the *Phenuiviridae* family within the *Bunyaviricites* class that affects ruminants, camelids, and humans. RVFV has caused multiple outbreaks in humans and livestock, most recently in 2025, when more than 400 laboratory-confirmed human cases of Rift Valley fever (RVF), with a case-fatality rate of approximately 10%, were reported in Mauritania and Senegal according to national authorities and the World Health Organization (WHO). Humans are primarily exposed to the virus through contact with contaminated tissues or blood during the slaughtering of RVFV-infected animals. While most human infections result in a self-limiting febrile illness, a significant proportion of cases develop neurological disorders or hemorrhagic fever, which can be fatal. RVFV infection is also associated with severe eye damage in up to 10% of reported cases, frequently leading to permanent blindness due to significant retinal scarring. Additionally, increasing evidence suggests that RVFV infection during pregnancy may harm the unborn fetus [1,2,3]. Currently, there are no therapeutics or vaccines available to prevent or treat human infections.

In animals, RVF primarily manifests as a severe, often fatal liver infection, especially in young animals. Additionally, epizootics in endemic regions such as Africa and the Arabian Peninsula are marked by abortion storms in pregnant herds. A wide range of mosquito species, particularly those within the genera *Aedes* and *Culex* [4,5], have been associated with the transmission of the virus. Given the widespread presence of competent vectors and susceptible animals in other regions of the world, it is anticipated that the virus will continue to expand its geographic range, driven by climate change and globalization [6].

The tri-segmented RVFV negative-sense RNA genome encodes a limited number of proteins, of which the nucleocapsid (N) protein, which protects the viral RNA, is considered the primary antigen of the virus, followed by the structural glycoproteins Gn and Gc. Currently, RVF diagnosis mainly involves reverse transcription polymerase chain reaction (RT-PCR) of blood and tissue samples and detection of RVFV-specific antibodies by ELISA and/or virus neutralization test (VNT), all to be performed in a specialized laboratory setting, equipped to handle highly pathogenic agents [7]. Pen-side tests offer a quick and cost-effective way to monitor and control RVF outbreaks more efficiently.

In 2019, a proof-of-concept lateral flow test (LFT) based on the RVFV N antigen was developed for rapid virus detection [8]. This technology was later refined and commercialized by Innovative Diagnostics, enabling field-ready antigen testing. More recently, a vertical flow immunoassay (VFI) based setup was described as well, though pending validation [9]. The first antibody-based LFT concept also emerged in 2019, describing IgG and IgM detection in sheep using Protein A capture [10]. In 2022, another study introduced an LFT for RVFV antibodies using an indirect binding format with Protein A-based capture and conjugates [11]. However, both described tests were performed using only a very limited number of sera and have not yet been compared against a gold-standard method.

Here, we report the development of a lateral flow test (LFT) to detect anti-RVFV-N antibodies employing a double-antigen format, alongside a corresponding double-antigen ELISA for comparative evaluation. The double-antigen approach is increasingly adopted in serological assay development and is particularly well suited for pathogens that infect multiple species and for which rapid, high-throughput detection is essential.

## 2. Materials and Methods

### 2.1. Antigen Production

The RVFV nucleoprotein (Strain 35/74, GenBank: AEF79994.1) was produced in *E-coli*. Briefly, the coding region was gene-synthesized (GenScript), cloned into a pET-30a vector, and fused with a C-terminal His-tag. Following IPTG induction, the protein was purified under native conditions according to the QIAexpressionist (Preparation of cleared *E. coli* lysates under native conditions and Batch purification of 6× His-tagged proteins from *E. coli* under native conditions).

### 2.2. Sample Panel

A serum sample set (Table 1) was collected from previously performed experimental RVF vaccination-challenge studies with sheep, goats, and cattle [12,13]. Sera from mock-vaccinated and wild-type virus-challenged animals were used. In addition, a human serum sample set provided by USAMRIID and obtained from a Phase 2 clinical trial with the MP-12 vaccine was used [14].

### 2.3. N-ELISA

The ID Screen^®^ Rift Valley Fever Competition Multi-species ELISA (Innovative Diagnostics, Grabels, France), as a commercial ELISA kit designed for the detection of anti-Rift Valley Fever (RVF) antibodies in serum or plasma from multiple species, including ruminants, horses, and dogs, was used according to the manufacturer’s instructions. The method is based on competition between N-binding antibodies present in test sera and a monoclonal antibody.

### 2.4. Double-Antigen ELISA Protocol

High-binding ELISA plates (Greiner, Kremsmünster, Austria, 655092) were coated with 100 µL per well of RVFV nucleocapsid protein (200 ng/mL in a 0.05 M carbonate-bicarbonate (pH 9.6) coating buffer) and incubated overnight at 4 °C. Plates were washed three times with 400 µL per well of PBS containing 0.05% Tween-20 (PBST) and blocked with 200 µL per well of blocking buffer (PBS + 0.05% Tween20 + 10% skimmed milk, Difco, Leeuwarden, The Netherlands, 232100) for 1 h at room temperature. Serum samples were diluted 1:5 in blocking buffer, and 50 µL was added per well for 1 h at room temperature. After washing three times with PBST, 50 µL of HRP-conjugated nucleocapsid antigen (prepared at a 1:4 ratio with an Abcam Lighting Link HRP Conjugation Kit (Abcam, Cambridge, UK), final concentration 500 ng/mL, diluted in blocking buffer) was added and incubated for 1 h at room temperature. Plates were washed three times with PBST, followed by the addition of 100 µL per well of TMB substrate (BioFX One Component HRP Microwell Substrate, Surmodics, Eden Prairie, MN, USA) and incubation for approximately 15 min at room temperature. The reaction was stopped with 50 µL per well of 0.5 M sulfuric acid (Merck), and absorbance was measured at 450 nm using a SpectraMax ABS Plus (Molecular Devices, San Jose, CA, USA). The cut-off for positivity was set at 3 times the standard deviation of negative samples.

### 2.5. LFT

**Antigen conjugation**. Detection N-antigen conjugate was prepared by coupling of antigen to carbon nanoparticles, as previously described by Willemsen et al. [15]. Briefly, N-antigen was buffer exchanged to borate buffer (BB) (5 mM, pH 8.8) using a desalting column and added to a freshly sonicated 0.2% (*w*/*v*) carbon nanoparticle suspension (CNP), at a concentration of 220 µg antigen per mL CNP. The preparation was stirred overnight at 4 °C on a magnetic stirrer. The preparation was washed thrice in wash buffer (BB, 5 mM, pH 8.8, containing 1% (*w*/*v*) skimmed milk protein (MilliPore, Burlington, MA, USA, 70166)) by sonication and centrifugation at 13,636× *g* (4 °C, 15 min). Finally, the preparation was resuspended to 0.2% CNP concentration in storage buffer (BB, 100 mM, pH 8.8, containing 1% (*w*/*v*) skimmed milk protein). The prepared conjugate was stored at 4 °C until further use.

**Test strip assembly*****.*** Following buffer exchange as described above, capture N-antigen (2 mg/mL) was sprayed on the nitrocellulose membrane (FF120HP, Cytiva, Marlborough, MA, USA, WH/10547001) using the Linomat 5 (Camag, Muttenz, Switzerland) at a rate of 4 µL/cm. A combined sample/conjugate pad was prepared by blocking the pads (8951, Ahlstrom, Helsinki, Finland) 3 times with blocking buffer (BB, 100 mM, pH 8.8, containing 1% (*w*/*v*) Bovine Serum Albumin (BSA, Sigma-Aldrich, Burlington, MA, USA, A3059)) and drying at 37 °C. The detection N-antigen conjugate was diluted 10× with conjugate dilution buffer (BB, 100 mM, pH 8.8 containing 1% (*w*/*v*) BSA and 3% (*w*/*v*) trehalose) and applied to the blocked sample/conjugate pad using an airjet (ZX1010 dispense system, Biodot, Irvine, CA, USA) at a rate of 20 µL/cm. Half sticks were assembled by combining the sprayed nitrocellulose membrane with an absorbent pad (222, Ahlstrom, Helsinki, Finland) on a lateral flow backing card (Kenosha Tapes, Amstelveen, The Netherlands). Full assays were assembled by combining the membrane with the sprayed sample/conjugate pad and an absorbent pad (270, Ahlstrom, Helsinki, Finland) on a backing. Cards were cut into 5 mm test strips using a cutter (CM4000, Bio Dot). Full assays were encased in a custom 3D-printed lateral flow cassette.

**Assay execution.** Assay execution was different for half-sticks and full assays. For the half sticks, the detection conjugate and serum sample were diluted in running buffer (BB, 100 mM, pH 8.8, containing 1% (*w*/*v*) skimmed milk and 0.05% (*v*/*v*) Tween 20), 100× and 50× in the final test volume, respectively. The serum dilution and conjugate dilution were mixed together in a 96-well plate, promptly followed by the test strip. The full assays were run by the addition of 50× diluted serum to the sample wells of the assays. For both the half sticks and full assays, the strips were allowed to dry and subsequently scanned with a flatbed scanner. The test signal was quantified with TotalLab Quant (v12.2), using the blanks for background subtraction. Data analysis was performed using GraphPad (v10.4) and Curve Expert Professional (v2.7).

## 3. Results

### 3.1. Double Antigen N-ELISA

**Figure 1:**
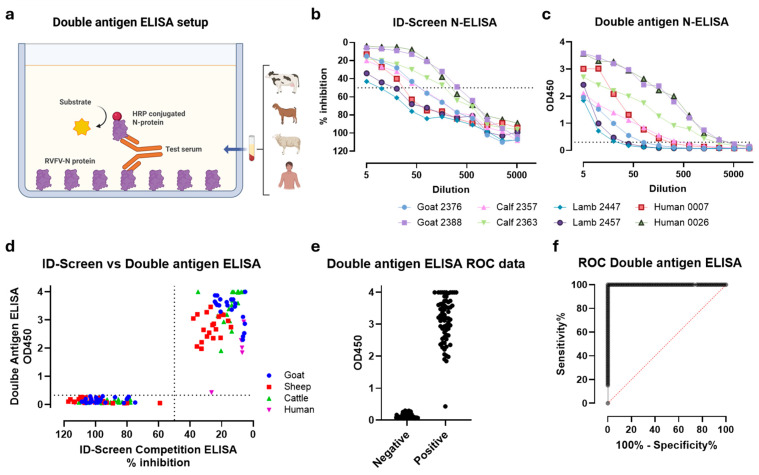
Double-antigen RVFV-N-ELISA. (**a**) Schematic representation of the double-antigen-based RVFV-N ELISA setup. Created in BioRender. Wichgers Schreur, P. (2026) https://BioRender.com/o7oaa0q (**b**) Serial dilution series of representative RVFV-N positive sera as tested with the ID-Screen competition ELISA according to the manufacturer’s instructions, and (**c**) the double antigen N-ELISA. Dashed lines (in **b**,**c**) represent the thresholds for positivity. For the ID-Screen ELISA, data is expressed as a percentage competition ratio of the optical densities of the sample and the optical densities of the negative control ([optical density sample/optical density negative control] × 100). Values below 40% are considered positive, those between 40% and 50% are considered doubtful, and those above 50% (dashed line) are considered negative. For the double-antigen ELISA, data are expressed as raw OD_450_ absorbance values, and a cut-off for positivity is defined as three times the standard deviation of negative samples. (**d**) Comparison of the double-antigen ELISA results with those obtained using the ID-Screen competition ELISA for an extended serum panel consisting of goat (*n* = 45), cow (*n* = 40), sheep (*n* = 40), and human (*n* = 10) negative and positive samples. Dashed lines indicate the positivity thresholds, consistent with panels (**b**,**c**). (**e**) Raw double-antigen ELISA data from the serum panel shown in (**d**), stratified into negative and positive groups, and used as input for panel (**f**). (**f**) ROC curve illustrating the relationship between sensitivity and specificity based on the dataset in (**e**).

With the ultimate aim to develop a robust LFT to detect anti-RVFV-N antibodies based on the double antigen setup, we initially developed a double antigen ELISA (Figure 1a). Using the ID Screen^®^ Rift Valley Fever Competition Multi-species commercial ELISA as the gold standard, the outcomes of a large panel of negative and positive sheep, goat, cattle, and human sera were compared. Initially, two positive sera per species were tested in dilution series in both assays. Of note, raw OD_450_ absorbance values are reported for the double-antigen ELISA, whereas results for the competition ELISA are expressed as relative percentage inhibition relative to the negative control, in accordance with the manufacturer’s instructions. The overall patterns were highly similar, with dilution series showing the highest results in the double-antigen test, also showing the highest results in the competition ELISA (Figure 1b,c). Following the comparative assessment of a larger serum panel (*n* = 135), the concordance between assays was confirmed. The results demonstrated complete agreement with the ID-Screen ELISA, achieving 100% sensitivity and 100% specificity, with a *p*-value < 0.0001 and an area under the ROC curve of 1.0 at the 95% confidence interval. With both specificity and sensitivity reaching 100%, the double antigen ELISA performs equivalently to the commercial competition ELISA for this dataset (Figure 1d–f).

### 3.2. Double Antigen N-LFT

**Figure 2:**
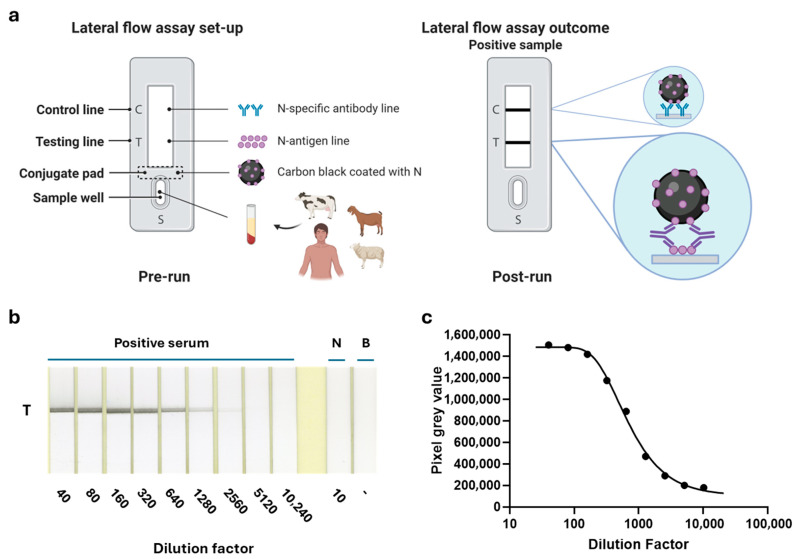
Double-antigen RVFV-N-LFT proof of principle. (**a**) Schematic representation of the double-antigen-based LFT-N setup. Created in BioRender. Wichgers Schreur, P. (2026) https://BioRender.com/sdn06us (**b**) A representative ovine N-positive serum was tested at different dilutions in the LFT (half sticks), and the test line indicated by (T). A negative serum (N) and a buffer-only (B) control were taken along. (**c**) Pixel gray values determined for the specific lines for each of the dilutions of the LFT presented in (**b**).

**Figure 3:**
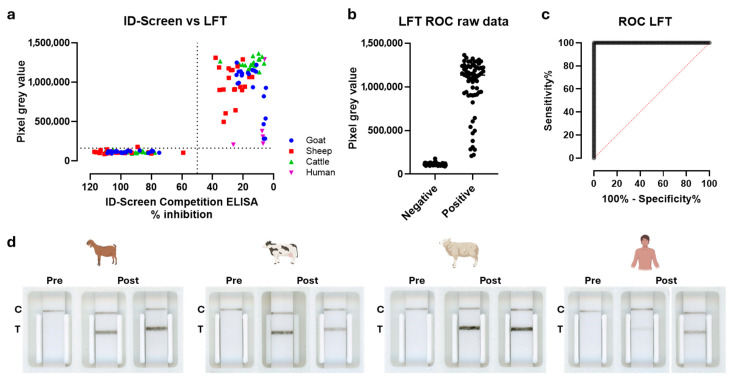
Qualification of double-antigen RVFV-N-LFT. (**a**) Comparison of the LFT results using half-strips in pixel gray values (PGV) with the results of the ID-Screen competition ELISA for an extended serum panel consisting of goat (*n* = 45), cow (*n* = 40), sheep (*n* = 40), and human (*n* = 10) samples. The competition ELISA data is expressed as a percentage competition ratio of the optical densities of the sample and the optical densities of the negative control ([optical density sample/optical density negative control] × 100). Values below 40% are considered positive, those between 40% and 50% are considered doubtful, and those above 50% (dashed line) are considered negative. The double-antigen LFT data is expressed as pixel gray values, and a cut-off for positivity is defined as three times the standard deviation of negative samples. (**b**) LFT data from the serum panel shown in (**a**), stratified into negative and positive groups and used as input for panel (**c**). (**c**) ROC curve (Wilson/Brown) illustrating the relationship between sensitivity and specificity based on the dataset in (**b**). (**d**) A representative panel of pre-immune and post-immune/challenge sera tested in full LFT assays with the control line indicated at (C) and the test line indicated with (T).

Confirming the double antigen setup is performing well in ELISA, we started to optimize the LFT based on a similar setup, though performed in a single step. The N-antigen was coupled to carbon nanoparticles and additionally immobilized on the nitrocellulose membrane (Figure 2a). This setup enables RVFV-specific antibodies in the test sera to bridge the N-proteins bound to both a carbon nanoparticle and the nitrocellulose fibers, forming a detectable complex. Following a series of optimization experiments using a small panel of positive and negative sera, a test setup was established that showed no detectable signal in negative sera, comparable to the running buffer background, and a distinct band in positive sera, which remained detectable even at high dilutions (Figure 2b). The LFT was subsequently tested with a broader panel of goat (*n* = 45), cow (*n* = 40), sheep (*n* = 40), and human (*n* = 10) immune sera and compared with the ID-Screen N-ELISA. The results demonstrated complete agreement with the ID-Screen ELISA, achieving 100% sensitivity and 100% specificity, with a *p*-value < 0.0001 and an area under the ROC curve (Wilson/Brown) of 1.0 at the 95% confidence interval. (Figure 3a–c). Importantly, in the LFT setup, no background signals were observed (both for negative sera and running buffer). Finally, some representative samples were also tested in a full assay setup as shown in Figure 3d. As expected, the control lines (C) were visible for each serum tested, whereas the test (T) line was visible in post-RVFV vaccination/challenge serum samples only.

## 4. Discussion

The developed RVFV double-antigen LFT and ELISA demonstrated performance highly comparable to the commercial ID-Screen^®^ ELISA using the same antigen, achieving complete diagnostic sensitivity and specificity at a 95% confidence interval. As a pen-side diagnostic, the LFT represents a valuable addition to efforts to combat the emerging RVFV threat, notably by substantially reducing the time from sample collection to result. A rapid turnaround is a critical advantage for effective management of this One Health pathogen across both veterinary and public health settings.

In both ELISA and LFT, we evaluated the double-antigen immunoassay approach, in which the same antigen is used for both capture and detection, enabling species- and immunoglobulin class-independent antibody detection [16]. This approach eliminates the need for species-specific secondary antibodies, enabling a single test to detect anti-RVFV antibodies in sera from any species. This greatly streamlines the development and implementation of multi-species serological diagnostics. The only limitation of this approach is its inability to distinguish between IgM and IgG, which may limit insights into the infection stage. However, in the absence of previous virus circulation, the detection of specific IgM antibodies still enables the identification of early infections. Future adaptations could incorporate selective binding elements or differential labeling to differentiate between early and late humoral responses, thereby enhancing their value for disease staging and epidemiological investigations.

In the commercial ID Screen^®^ RVF ELISA, a monoclonal antibody is used that binds the N-protein. Only serum antibodies that bind to the same, or to an overlapping or adjacent epitope, thereby sterically hindering the MAb from binding, will be detected in the test. However, antibodies that bind to different epitopes will not compete in the test and will remain undetected. In contrast, these antibodies will be detected in immunoassays based on the double antigen approach. This potential added value did not become apparent in our analysis; both assays worked very well. However, it is likely that the monoclonal antibody used in the commercial test is directed to an immuno-dominant epitope on the N protein, thereby minimizing the difference with outcomes of tests based on the double antigen approach.

For RVFV, the nucleocapsid (N) protein is considered the most immunogenic antigen, and antibodies against it persist for an extended period post-infection, making it an ideal target for serological assays. The N protein can be produced in high yields using recombinant expression systems, which helps reduce the cost of goods for lateral flow tests. Moreover, because some RVFV vaccines exclude the N protein, an N-based LFT can serve as a DIVA (differentiating infected from vaccinated animals) assay, which is urgently needed [17]. Conversely, other vaccines omit the NSs protein, suggesting that a multi-analyte test detecting both N- and NSs-specific antibodies could also function as a DIVA tool. Current evidence indicates that RVFV N protein can be considered a specific diagnostic target; only very few antigenic cross-reactions have been observed between RVFV and other phleboviruses in diagnostic assays, which may even be related to other antigens [18]. However, full validation using field sera, sera specific to related phleboviruses, and sera from acute-phase cases, especially of human origin, is still pending and remains a key limitation of this study. Progress in this direction is mainly constrained by the limited availability of well-characterized sample sets.

For this study, we selected carbon nanoparticles because they are cost-effective and provide a stronger visual contrast and greater chemical stability than gold nanoparticles, resulting in an easily interpretable signal in lateral flow formats. Additionally, the sensitivity of carbon nanoparticle-based lateral flow assays has been compared to that of gold-based assays and turned out to be superior [19,20]. The superior sensitivity of carbon nanoparticles also benefits future assay expansion [21]. Although the current test is specifically designed for RVFV detection, the platform can be readily adapted to a multi-analyte format, enabling the simultaneous detection of multiple pathogens of veterinary and zoonotic importance in a single test. Notably, Godarzi et al. reported the development of a carbon nanoparticle-based microarray lateral flow immunoassay to detect West Nile virus and Usutu Virus-specific antibodies in serum of various species [19,22]. This multiplex LFT, applying multiple spots instead of lines, was combined with a real-time kinetic video reader, providing enhanced analytical performance and enabling kinetic and/or fully quantitative result interpretation.

In addition to multiplexing, the use of whole blood, instead of serum, as sample input would also be favorable under field conditions where sample processing is challenging. Whole blood can impair capillary flow due to blockage of the nitrocellulose membrane by erythrocytes and white blood cells, introduce matrix effects, and reduce the visibility of test lines upon lysis of erythrocytes. To overcome these issues, blood separation membranes/pads have been introduced in LFTs to entrap cells, whereas plasma can freely move into the nitrocellulose membrane [23,24]. Adoption of such measures will substantially improve the field applicability and robustness of RVFV diagnostics, particularly in resource-limited settings.

In conclusion, we report the qualification of a multispecies double-antigen ELISA and LFT using sera from sheep, cattle, goats, and humans, and benchmarked against a commercial competition ELISA. Both the LFT and the double-antigen ELISA demonstrated high sensitivity and specificity, underscoring their strong diagnostic performance. Collectively, these advances bring RVFV-specific pen-side testing substantially closer to practical field implementation.

## Figures and Tables

**Table 1:** Serum panel.

Species Origin	RVFV Strain	Time Post Infection	Ref.
Sheep	RVFV-35/74	0 and 3 weeks	[13]
Goat	RVFV-35/74	0 and 3 weeks	[12]
Cattle	RVFV-35/74	0 and 3 weeks	[12]
Human	RVFV MP12	0 and ~3 years	[14]

## Data Availability

Data is contained within the article.
